# The Esophageal Adenocarcinoma Epidemic Has Reached Hungary: A Multicenter, Cross-Sectional Study

**DOI:** 10.3389/fonc.2020.541794

**Published:** 2020-12-23

**Authors:** Benedek Tinusz, László Botond Szapáry, Bence Paládi, András Papp, Barna Bogner, Ivett Hegedűs, Szabolcs Bellyei, Áron Vincze, Jenő Solt, Tamás Micsik, Veronika Dunás-Varga, Eszter Pályu, Tamás Vass, Tamás Schnabel, Nelli Farkas, Péter Hegyi, Aaron P. Thrift, Bálint Erőss

**Affiliations:** ^1^Medical School, Institute for Translational Medicine and Szentágothai Research Center, University of Pécs, Pécs, Hungary; ^2^Department of Surgery, University of Pécs, Pécs, Hungary; ^3^Department of Pathology, Medical School, University of Pécs, Pécs, Hungary; ^4^Department of Oncotherapy, Medical School, University of Pécs, Pécs, Hungary; ^5^Department of Gastroenterology, 1st Department of Medicine, Medical School, University of Pécs, Pécs, Hungary; ^6^1st Department of Pathology and Experimental Cancer Research, Semmelweis University, Budapest, Hungary; ^7^1st Department of Internal Medicine, Szent György University Teaching Hospital of Fejér County, Székesfehérvár, Hungary; ^8^2nd Department of Internal Medicine, University of Debrecen, Debrecen, Hungary; ^9^1st Department of Surgery, Semmelweis University, Budapest, Hungary; ^10^Department of Gastroenterology, Saint John’s Hospital, Budapest, Hungary; ^11^Medical School, Institute of Bioanalysis, University of Pécs, Pécs, Hungary; ^12^Department of Medicine and Dan L. Duncan Comprehensive Cancer Center, Baylor College of Medicine, Houston, TX, United States

**Keywords:** epidemiology, incidence, esophageal adenocarcinoma, esophagus, cancer, esophageal cancer, oncology

## Abstract

**Background:**

The epidemiology of esophageal cancer has changed dramatically over the past 4 decades in many Western populations. We aimed to understand the Hungarian epidemiologic trends of esophageal squamous cell cancer (SCC) and adenocarcinoma (AC).

**Methods:**

We performed a cross-sectional study using data from esophageal cancer patients diagnosed between 1992 and 2018 at eight tertiary referral centers in four major cities of Hungary. We retrospectively identified cases in the electronic databases of each center and collected data on gender, age at diagnosis, year of diagnosis, specialty of the origin center, histological type, and localization of the tumor. Patients were grouped based on the two main histological types: AC or SCC. For statistical analysis, we used linear regression models, chi-square tests, and independent sample t tests.

**Results:**

We extracted data on 3,283 patients with esophageal cancer. Of these, 2,632 were diagnosed with either of the two main histological types; 737 had AC and 1,895 SCC. There was no significant difference in the gender ratio of the patients between AC and SCC (80.1 *vs* 81.8% males, respectively; p = 0.261). The relative incidence of AC increased over the years (p < 0.001, b = 1.19 CI: 0.84–1.54). AC patients were older at diagnosis than SCC patients (64.37 ± 11.59 *vs* 60.30 ± 10.07 years, p < 0.001). The age of patients at the diagnosis of primary esophageal cancer increased over time (p < 0.001, R = 0.119).

**Conclusions:**

The rapid increase in the relative incidence of AC and simultaneous decrease of the relative incidence of SCC suggest that this well-established Western phenomenon is also present in Hungary.

## Highlights

Our retrospective cross-sectional study aimed to confirm or disprove the presence of the so-called “esophageal adenocarcinoma epidemic” in Hungary. We included over 2,500 esophageal cancer patients diagnosed between 1992 and 2018. Our results indicate that the relative incidence of adenocarcinoma is increasing over the years while that of squamous cell cancer is decreasing simultaneously.

## Introduction

Esophageal cancer is the seventh most common cancer worldwide with an estimated 572,000 new cases diagnosed yearly. Simultaneously, over 508,000 patients die due to this disease each year (1), which makes esophageal cancers the sixth leading cause of cancer-related mortality (2).

The two major histological types of esophageal cancer are adenocarcinoma (AC) and squamous cell cancer (SCC). The worldwide incidence of SCC is traditionally higher than that of AC (398,000 and 52,000 new cases of SCC and AC in 2012, respectively) (3). Recently, a major shift has been reported regarding the ratio of the two histological types in several developed countries of North America, Oceania, Western and Northern Europe. In these countries, the incidence of AC has been increasing along with a simultaneous decrease in the incidence of SCC (4). As a result, AC is now the most common form of esophageal cancer in these populations. A possible explanation for this phenomenon (labeled as the “esophageal adenocarcinoma epidemic”), is the difference between risk factors of AC and SCC and their changing prevalence in high-income countries (4–7).

The scarce and incomplete epidemiologic data on esophageal cancer in Hungary and Eastern Europe, especially on the ratio of histological types of esophageal cancers make it unclear whether the aforementioned epidemiological trend detected in western countries also affects this region.

Our study aimed to systematically gather and analyze detailed epidemiological data on esophageal cancers in Hungary with the main hypothesis that, similarly to other developed countries, the incidence of esophageal AC is increasing while the incidence of SCC is decreasing.

## Materials and Methods

### The EAGLE-R Study

The EAGLE-R (EsophAGeal cancers and precancerous LEsions in Hungary—a Retrospective, epidemiological investigation) study was organized by the Hungarian Esophagus Study Group with the purpose of retrospectively gathering data from esophageal cancer patients. The study protocol was approved by the Hungarian Scientific and Research Ethics Committee of the Medical Research Council (registration number 65414-2/2017/EKU). The procedures followed were in accordance with the ethical standards of the Helsinki Declaration of the World Medical Association. A total of eight tertiary referral centers in four major cities of Hungary (Budapest, Debrecen, Pécs, Székesfehérvár) contributed patient data to the analysis.

### Inclusion Criteria and Identification

We included all patients diagnosed with esophageal malignancies, confirmed by endoscopy and histology. Statistical analysis was only performed on patients that belonged to the two main histological subgroups (AC and SCC). Patients were identified by searching for “International Classification of Diseases” codes corresponding to esophageal cancer (C15*) in the electronic medical database of each center. The time period examined varied by centers and depended on the time of implementation of such databases (see **Supplementary 1**). We used social security numbers to filter out duplicates. After finishing data collection, these numbers were discarded to ensure anonymity.

### Data Collection

The following data were collected from each patient: year of diagnosis, age at the time of diagnosis, gender, the localization of the tumor in the esophagus, and the histological type of the tumor. The specialty of the center of origin (i.e. surgery or gastroenterology) and city of origin were also recorded. Data were obtained from clinic letters and other relevant documents detailing past medical history, gastroscopic, histological, and imaging examinations. The esophageal localization was categorized as upper, middle, or lower third, based on the affected segment of the esophagus. Where the location of the tumor was documented in centimeters (cm) from the incisors, then 18–25, 25–32, and more distal than 32 cm corresponds to upper, middle, and distal cancers, respectively. In those instances where the tumor affected two neighboring segments, the localization was described as upper-middle or lower-middle in our data sheet.

### Grouping

Patients were grouped together based on the two most prominent histological types of esophageal cancer: AC and SCC. Data on the histology of each tumor were obtained by checking individual histological reports rather than searching for histology codes.

### Outcomes

Our primary outcome was the relative incidence of AC over the years compared to that of SCC. Since our analysis was limited to just a part of the Hungarian population, we were unable to determine the true incidence of esophageal cancers for the whole population. Rather, the ratio of esophageal AC and SCC (i.e. relative incidence) was calculated within the combined group.

The secondary outcomes included the mean age at the time diagnosis and its change over the years. In addition, we looked at the rate of histological types and gender ratio based on the specialty of the center of origin. We analyzed the distribution of genders between histological subgroups as well as the Hungarian population and our cohort of patients. The esophageal localization of the tumor was also compared between the two main groups.

### Data Synthesis

Descriptive statistical tools were used to describe the basic characteristics of the study populations. In case of continuous variables independent sample t-tests were used to observe differences between groups. In case of categorical outcomes Chi-square tests or proportion tests were applied. To detect trends or associations, linear regression analyses were used, we calculated the slope (b) of the trendline with 95% confidence interval. To eliminate the effect of population-wide aging, we constructed the difference between the age at the diagnosis and the average life expectancy at birth, and we used these indices for statistical calculation.

All analyses were conducted using IBM-SPSS Statistical Software version 25 (IBM Corporation, Armonk, NY, USA).

Data on gender distribution of the Hungarian population and yearly average life expectancy at birth were obtained from the website of the Hungarian Central Statistical Office (8).

## Results

### Patient Numbers and Data Quality

Data from a total of 3,283 patients diagnosed with esophageal malignancies between 1992 and 2018 were extracted from the electronic databases of the eight centers involved. We analyzed data originating from four major Hungarian cities including Budapest (4 centers, 1,128 patients), Debrecen (1 center, 896 patients), Pécs (3 centers, 938 patients), and Székesfehérvár (1 center, 321 patients). For a detailed list on the number of patients and the examined period in each center, see **Supplementary 1**. A total of 2,982 patients had primary esophageal cancer, of whom 2,632 patients had either AC (737) or SCC (1,895). For the full distribution and general characteristics of the 3,283 patients involved in the study, see **Table 1**. The average completeness of data for the analyzed group was 99.83% (see **Supplementary 2**).

**Table 1:** General characteristics of the full database.

Characteristic	N (percentage of total)
Total number	3,283 (100.0%)
Male	2,608 (80.1%)
Histological type	
SCC	1,895 (57.7%)
AC	737 (22.4%)
Other primary esophageal cancer	79 (2.4%)
No data (but primary esophageal cancer)	271 (8.3%)
Metastasis or infiltration by non-esophageal cancer	217 (6.6%)
Malignancy not proven—excluded from analysis	84 (2.6%)
Mean age	62.0 (± 11.0^1^)

SCC, squamous cell cancer; AC, adenocarcinoma; ^1^Standard deviation.

### Primary Outcome

#### AC Is Becoming More Common Over Time

**Figure 1:**
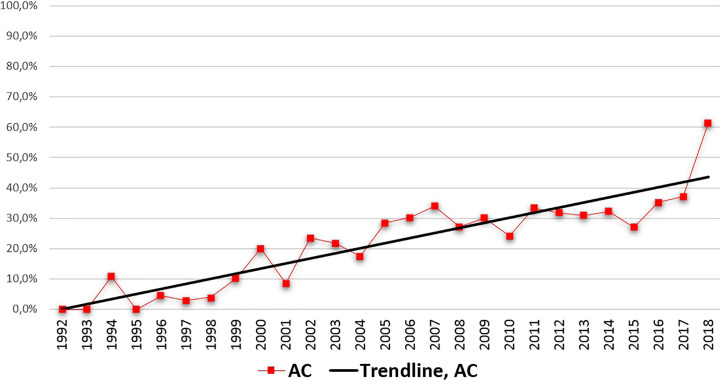
Relative incidence of adenocarcinoma over the years. Linear regression, p < 0.001, b = 1.189, confidence interval (CI): 0.837–1.541.

We found that the relative incidence of AC had been increasing significantly in the examined period, compared to the relative incidence of SCC (p < 0.001, b = 1.19 CI: 0.84–1.54, **Figure 1**). In 2018, the relative incidence of AC overtook that of SCC with AC accounting for 61.3% of patients in the analyzed group, however, this value is likely to be an outlier due to the small patient number that year. As an example, according to our database, within the first 10 years of the examined period (1992–2001), the average percentage of SCC and AC was 93.9 and 6.1% respectively, as compared to the last 10 years of the examined period (2009–2018), where SCC and AC accounted for an average of 65.7% and 34.3%, respectively. For a detailed breakdown of relative incidences over the years, see **Supplementary 3**.

### Secondary Outcomes

#### AC Is Diagnosed in Older Patients

Patients in the AC group were diagnosed at a significantly higher age, compared to patients in the SCC group (mean age, 64.37 ± 11.59 and 60.30 ± 10.07 years in the AC and SCC groups, respectively; p < 0.001; **Table 2**). The average age at diagnosis in the analyzed group had been significantly increasing over the years, and the significance was still present when correcting for average life expectancy at birth (R = 0.119; p < 0.001). This correlation was also present when looking at the SCC group only (R = 0.132; p < 0.001), but not when analyzing the AC group (R = 0.016; p < 0.671). **Supplementary 4** contains further data on the age at diagnosis in an annual breakdown.

**Table 2:** Characteristics and results from the analyzed group.

	Total (% of column total)	SCC group (% of column total)	AC group (% of column total)	p-value
Number of patients	2,632 (100.0%)	1,895 (100.0%)	737 (100%)	
Male gender	2142 (81.38%)	1,550 (81.79%)	592 (80.33%)	0.261^2^
Mean age at diagnosis	61.44 (10.68^1^)	60.30 (10.07^1^)	64.37 (11.59^1^)	<0.001^3^
Localization				
Upper	399 (15.16%)	389 (20.53%)	10 (1.36%)	<0.001^2^
Middle	710 (26.98%)	674 (35.57%)	36 (4.88%)	
Lower	1,125 (42.74%)	492 (25.96%)	633 (85.89%)	
Lower-middle	158 (6.00%)	125 (6.60%)	33 (4.48%)	
Upper-middle	81 (3.08%)	80 (4.22%)	1 (0.14%)	
No data	159 (6.04%)	135 (7.12%)	24 (3.26%)	

SCC, squamous cellular cancer; AC, adenocarcinoma; ^1^Standard deviation; ^2^Chi-squared test; ^3^One sample T-test; p values always characterize the difference between AC and SCC groups.

#### AC Is More Common in Surgical Centers

The ratio of AC is higher in those patients who visited a surgical center, compared to those who originate from a gastroenterology department. The proportion of AC patients in surgical and gastroenterology centers was 34.53% (394 out of 1,141 patients) and 19.57% (73 out of 373 patients), respectively (p < 0.001; **Supplementary 5**).

#### Male Predominance Characterizes Both Groups

We found no significant difference in the gender distribution between the two groups. The proportion of male patients in the SCC and AC groups was 81.79% (1,550 out of 1,895 patients) and 80.33% (592 out of 737 patients), respectively (p = 0.261; **Table 2**). There was a significant difference in the gender distribution of our analyzed cohort and the whole population of Hungary, with the proportion of males being much higher in the former (80.38 and 47.80%, respectively; p < 0.001). For a yearly breakdown of gender distribution in the analyzed group, see **Supplementary 6**.

#### AC Originates More Commonly From the Distal Esophagus

Our analysis showed that AC develops more often at the distal part of the esophagus, compared to SCCs. The percentage of tumors originating from the lower third of the esophagus in the SCC and AC groups are 25.96 and 85.89%, respectively (p < 0.001, **Table 2**).

## Discussion

### General Discussion

The results of our study indicate that the relative incidence of AC in Hungary is increasing over the years with the simultaneous decrease in the relative incidence of SCC. Additionally, the age at which AC patients are getting diagnosed increases over the years. We found that among patients who visit a surgical center, the ratio of AC is higher than that of SCC. Moreover, our study also proved some well-known facts, such as male dominance in both groups, and the fact that AC originates more commonly from the distal esophagus than SCC.

Data on the incidence of esophageal cancers in Hungary are scarce and incomplete. A total yearly incidence of around 800 is reported by various sources, with an average 5-year survival of 15–34% (1, 9). Another study found a significant decrease in the age-standardized mortality rates (ASMR) of esophageal cancer between 1998 and 2012, which was preceded by a significant increase indicated by several other articles (10, 11). An ASMR of 7.83 per 100,000 people per year was reported for 2012 (12). Even with this trend accounted for, the Hungarian ASMRs of esophageal cancer were still the highest among member countries of the EU within two age groups in the 2000–2009 period (13).

To our best knowledge, no country-wide study has reported data on the histological distribution of cases. A single-center study on 451 total patients diagnosed with esophageal or cardia cancers between 1993 and 2003 did not find an increase in the proportion of ACs (proportion of SCC and AC were 93 and 4%, respectively) and concluded that this histological type is still infrequent in Hungary (14).

Our finding regarding the increase of esophageal AC incidence contradicts the conclusion of the aforementioned retrospective study and is in line with several epidemiological investigations not only from western countries (15, 16), but Hungary’s geographical neighbors, such as Croatia and Slovakia as well (4, 7, 17, 18).

The increase in the relative incidence of AC is contributed to the rise of its risk factors in developed countries (4–7, 19). These include obesity, smoking, gastroesophageal reflux disease (GERD), and the consequent Barrett’s esophagus. All mentioned risk factors are linked with urban lifestyle (5, 20, 21).

Similarly to other developed countries, the prevalence of obesity has been on the rise in Hungary for the past 30 years, with the age-standardized prevalence of obesity in 2013 estimated to be 21.7 and 24.7% in adult males and females, respectively (22). In addition to being an independent risk factor for the development of esophageal AC, obesity also promotes the development of GERD (23, 24).

A retrospective cohort study with over 100,000 participants showed no significant increase in the prevalence of regular smoking between 1982 and 2013, with the prevalence of regular smokers in the 2006–2013 period estimated to be 34.7 and 14.9% in adult males and females, respectively (25).

No nationwide data is available on the prevalence of Barrett’s esophagus and GERD in Hungary, therefore a possible increase in the prevalence of these diseases cannot be excluded as an underlying cause behind the rise in the relative incidence of AC. Chronic *H. pylori* infection is inversely associated with the risk of developing GERD, most likely through the reduction of gastric acid production due to chronic atrophic gastritis caused by the bacteria (26). A single-center retrospective study on 4,627 patients demonstrated a significant reduction of *H. pylori* infections in Budapest (27). This trend matches the results from other developed countries and may explain part of the increasing AC incidence (28).

Our finding that esophageal cancer is getting diagnosed at a higher age may partially be explained by the increasing proportion of AC and the significantly older age at diagnosis in this group. There are no obvious explanations as to why this increase is present in the SCC group, and this could be the main question of a future study.

We found that the ratio of AC is significantly higher in surgical centers, compared to gastroenterology centers, which suggests that patients with AC are more likely to be eligible for the consideration of curative resectional surgery due to the predominantly distal localization of the tumor.

Concerning the delay of around 3 decades between Hungary and modern western countries in terms of the increase in the incidence of esophageal AC, we hypothesize that the factors associated with the so-called “Western lifestyle” and their delayed appearance in Hungary may lie in the background. These include processed and fast food consumption and the consequent occurrence of obesity, which became prevalent much earlier in the US (29), compared to Hungary (30). In our country, these eating habits started gaining popularity after the fall of the Berlin Wall in 1990, when capitalistic ideologies and customs started replacing the old communist system. For example, the prevalence of overweight people in 1990 was 50 and 13% in the USA and in Hungary, respectively, with this number further increasing over the years in both countries (31, 32). In addition, the higher prevalence of risk factors associated with esophageal SCC in Hungary could also explain why SCC is still the dominant histological type. One such factor is alcohol consumption, which is traditionally higher in Hungary compared to the US (33, 34).

### Representativeness

Considering we were only able to gather data on the minority of the new cases each year, it is of paramount importance to prove the representativeness of our data. Unfortunately, the lack of nation-wide studies makes this process difficult due to the lack of large Hungarian patient populations to compare with.

However, the differences we found between the two groups in terms of the distribution of genders and the localization in the esophagus are already well-explained and well-known facts (5, 20). The importance of proving these associations in our cohort lies not so much in their novelty, rather in the validity they provide to our database and other findings.

### Limitations

While conducting our study, we came across several hurdles that could potentially impair the strength of our findings.

Our initial aim with the EAGLE-R study was to gather and analyze data on a much broader variety of outcomes, such as detailed information about the past medical history including risk factors, previous diseases, and therapeutic procedures. However, we were confronted with the difficulties of retrospective data collection, meaning that only a fraction of the planned parameters was available to extract data on with sufficiently high quality.

The fact that no data were collected on the risk factors of esophageal cancer makes certain findings of our study difficult to explain. One such example is that epidemiologic data on the prevalence of Barrett’s esophagus would give a possible explanation for the rise of AC incidence over the years. We can only suspect that there may be an unnoticed elevation of Barrett’s prevalence partly driving the rise in the prevalence of esophageal AC.

Another point is that the introduction of classification systems, such as Siewert’s classification, has improved the distinction of the distal esophageal cancers from gastric cardia tumors over the years. The fact that we were unable to obtain information on the number of cases with cardia involvement, makes it possible that some centers classifying these as AC of the esophagus may have had an impact on our results.

Another major limitation is that we only had access to a relatively small percentage of the total number of new cases of esophageal cancers yearly; consequently, only relative incidence (of AC and SCC) could be calculated using this limited amount of data.

Moreover, the fact that the year of implementing electronic databases varied from center to center meant that each center had a different period they provided patients from. This inconsistency of patient sources within the 26-year period should also be considered when interpreting our results.

The unusually steep increase in the relative incidence of AC in 2018, compared to the previous years could be contributed to the low patient number that year. This outlier is likely to be the consequence of the total number of patients being 75 (from three centers) in 2018. In comparison, this number was 169 (from six centers) in 2017 (**Supplementaries 1** and **3**).

### Conclusions

#### Implications for Research

Parallel to providing considerable data on the epidemiology of esophageal cancers in Hungary, our study raises several questions. Due to the drawbacks of retrospective data collection, the explanation for the epidemiological trends remains uncertain. We launched the Hungarian esophageal cancer registry, a prospective analysis with the aim of shedding light to unanswered questions using its long-term follow-up and detailed questionnaire on risk factors, therapeutic modalities, and quality of life changes.

#### Implications for Practice

The main conclusion of our study is that the “esophageal adenocarcinoma epidemic,” which has mostly been described in high-income western countries, can also be detected in Hungary. This fact can have serious implications for clinical practice, considering esophageal AC (while still a tumor with poor prognosis) is generally less invasive and metastasizes at a later stage, compared to SCC. Therefore, we predict a slight increase in the ratio of esophageal cancers where curative therapy can be applied, and, parallel to this, a slight drop in terms of mortality. Moreover, with the increasing ratio of AC, we expect the importance and value of Barrett’s esophagus surveillance endoscopies to rise.

## Data Availability Statement

The datasets generated for this study are available on request to the corresponding author.

## Ethics Statement

The studies involving human participants were reviewed and approved by Hungarian Scientific and Research Ethics Committee of the Medical Research Council (registration number 65414-2/2017/EKU). Written informed consent for participation was not required for this study in accordance with the national legislation and the institutional requirements.

## Author Contributions

BE and PH conceptualized and designed the study in cooperation with BT. BT, LS, and BP constructed the forms to be filled with patient data. BT, BE, SB, and ÁV contributed to the ethical approval process of the study. BT, BP, and LS performed the data extraction from multiple centers. AP, BB, IH, VD-V, EP, TV, and TS gathered patient data from their respective centers. BT and BE wrote the article. PH and BE supervised the study. TM, AT, and JS provided valuable feedback after critically reviewing the first drafts of the manuscript. NF carried out the statistical analysis. All authors contributed to the article and approved the submitted version.

## Funding

This study was funded by “GINOP-2.3.2-15-2016-00048 - STAY ALIVE” co-financed by the European Union (European Regional Development Fund) within the framework of Programme Széchenyi 2020, and by Human Resources Development Operational Programme Grant, Grant Number: EFOP 3.6.2‐16‐2017‐00006 – LIVE LONGER which is co-financed by the European Union (European Regional Development Fund) within the framework of Programme Széchenyi 2020.

## Conflict of Interest

The authors declare that the research was conducted in the absence of any commercial or financial relationships that could be construed as a potential conflict of interest.

## Supplementary Material

Supplementary 1 Data contribution of centers.

Supplementary 2 Completeness of data for the analyzed group.

Supplementary 3 Detailed data on the relative incidence of the two main histological types.

Supplementary 4 Detailed data on the age at diagnosis of the two main histological types.

Supplementary 5 Distribution of patients based on center of origin.

Supplementary 6 Ratio of males in the two main histological groups.

Supplementary 7 Distribution of primary esophageal cancers by year.
