# Supplementary material for: The Esophageal Adenocarcinoma Epidemic Has Reached Hungary: A Multicenter, Cross-Sectional Study
Source: Front Oncol. 2020 Dec 23;10:541794. doi: 10.3389/fonc.2020.541794 (PMC7786275; doi:10.3389/fonc.2020.541794)
Supplement: Supplementary 1 — Data contribution of centers. [file Table_1.docx]

**Supplementary 1.:** Data contribution of centers

| **City** | **Center** | **Number of patients** | **Time period** |
| --- | --- | --- | --- |
| Budapest | 1st Department of Pathology and Experimental Cancer Research, Semmelweis University | 216 | 1998-2017 |
|  | 1st Department of Surgery, Semmelweis University | 696 | 2000-2018 |
|  | Department of Gastroenterology, Saint John’s Hospital | 216 | 2004-2018 |
| Debrecen | 2nd Department of Internal Medicine, University of Debrecen | 896 | 1995-2017 |
| Pécs | 1st Department of Medicine, University of Pécs | 142 | 2005-2017 |
|  | Department of Pathology, University of Pécs | 272 | 1999-2016 |
|  | Department of Surgery, University of Pécs | 524 | 1992-2013 |
| Székesfehérvár | 1st Department of Internal Medicine, Szent György University Teaching Hospital of Fejér County | 321 | 1997-2018 |
| **ALTOGETHER** |  | **3283** | **1992-2018** |

**Supplementary 2.:** Completeness of data for the analyzed group

|  | **Total number of patients** | **Number of patients with available data** | **Percentage of total** |
| --- | --- | --- | --- |
| Gender | 2632 | 2612 | 99.24% |
| Specialty of the center of origin | 2632 | 2632 | 100.00% |
| City of origin | 2632 | 2632 | 100.00% |
| Year of diagnosis | 2632 | 2632 | 100.00% |
| Age at diagnosis | 2632 | 2632 | 100.00% |
| Histological subtype | 2632 | 2632 | 100.00% |
| Localization in the esophagus | 2632 | 2632 | 100.00% |
| Year of birth | 2632 | 2632 | 100.00% |
| Life expectancy at birth | 2632 | 2612 | 99.24% |
| **AVERAGE UPLOADED DATA** |  |  | **99.83%** |

**Supplementary 3.:** Detailed data on the relative incidence of the two main histological types (SCC: squamous cell cancer, AC: adenocarcinoma)

| **Year** | **Total number of patients** | **SCC**  **(% of total)** | **AC**  **(% of total)** |
| --- | --- | --- | --- |
| 1992 | 18 | 100.0% | 0.0% |
| 1993 | 21 | 100.0% | 0.0% |
| 1994 | 37 | 89.2% | 10.8% |
| 1995 | 16 | 100.0% | 0.0% |
| 1996 | 22 | 95.5% | 4.5% |
| 1997 | 35 | 97.1% | 2.9% |
| 1998 | 51 | 96.1% | 3.9% |
| 1999 | 59 | 89.8% | 10.2% |
| 2000 | 40 | 80.0% | 20.0% |
| 2001 | 47 | 91.5% | 8.5% |
| 2002 | 64 | 76.6% | 23.4% |
| 2003 | 55 | 78.2% | 21.8% |
| 2004 | 57 | 82.5% | 17.5% |
| 2005 | 70 | 71.4% | 28.6% |
| 2006 | 76 | 69.7% | 30.3% |
| 2007 | 53 | 66.0% | 34.0% |
| 2008 | 177 | 72.9% | 27.1% |
| 2009 | 166 | 69.9% | 30.1% |
| 2010 | 195 | 75.9% | 24.1% |
| 2011 | 189 | 66.7% | 33.3% |
| 2012 | 201 | 68.2% | 31.8% |
| 2013 | 184 | 69.0% | 31.0% |
| 2014 | 199 | 67.8% | 32.2% |
| 2015 | 174 | 73.0% | 27.0% |
| 2016 | 182 | 64.8% | 35.2% |
| 2017 | 169 | 62.7% | 37.3% |
| 2018 | 75 | 38.7% | 61.3% |

**Supplementary 4.:** Detailed data on the age at diagnosis of the two main histological types (SCC: squamous cell cancer, AC: adenocarcinoma)

| **Year** | **Mean age at diagnosis (overall, years±SD)** | **Mean age at diagnosis (SCC years±SD)** | **Mean age at diagnosis (AC years±SD)** | |
| --- | --- | --- | --- | --- |
| 1992 | 49.88 (±11.12) | 49.89 (±11.12) | | No data |
| 1993 | 53.90 (±9.16) | 53.90 (±9.16) | | No data |
| 1994 | 47.57 (±11.90) | 47.76 (±12.21) | | 46.00 (±10.17) |
| 1995 | 43.63 (±10.85) | 43.63 (±10.85) | | No data |
| 1996 | 46.45 (±13.31) | 46.71 (±13.58) | | 41.00 (±0.00) |
| 1997 | 55.31 (±10.75) | 55.21 (±10.89) | | 59.00 (±0.00) |
| 1998 | 56.88 (±8.49) | 56.29 (±8.09) | | 71.50 (±4.95) |
| 1999 | 58.25 (±10.47) | 57.74 (±10.49) | | 62.83 (±10.01) |
| 2000 | 57.03 (±12.30) | 56.00 (±12.64) | | 61.13 (±10.48) |
| 2001 | 58.00 (±11.34) | 57.58 (±11.21) | | 62.50 (±13.53) |
| 2002 | 59.41 (±10.28) | 57.00 (±8.92) | | 67.27 (±10.79) |
| 2003 | 58.75 (±9.94) | 57.02 (±8.80) | | 64.92 (±11.67) |
| 2004 | 59.72 (±8.19) | 59.19 (±7.98) | | 62.20 (±9.14) |
| 2005 | 60.06 (±10.80) | 57.44 (±8.85) | | 66.60 (±12.60) |
| 2006 | 60.58 (±11.73) | 59.70 (±11.71) | | 62.61 (±11.78) |
| 2007 | 59.23 (±9.09) | 58.74 (±6.81) | | 60.17 (±12.59) |
| 2008 | 59.37 (±9.43) | 59.19 (±8.31) | | 59.85 (±12.02) |
| 2009 | 60.07 (±9.90) | 58.95 (±8.76) | | 62.66 (±11.84) |
| 2010 | 62.36 (±10.31) | 61.35 (±9.13) | | 65.55 (±12.98) |
| 2011 | 61.98 (±10.29) | 62.55 (±9.27) | | 60.84 (±12.07) |
| 2012 | 62.58 (±9.35) | 62.50 (±8.58) | | 62.73 (±10.89) |
| 2013 | 63.94 (±9.90) | 63.24 (±9.03) | | 65.49 (±11.53) |
| 2014 | 63.69 (±9.28) | 63.26 (±8.69) | | 64.59 (±10.43) |
| 2015 | 64.90 (±10.02) | 64.09 (±9.11) | | 67.09 (±12.00) |
| 2016 | 65.56 (±9.24) | 64.27 (±8.46) | | 67.94 (±10.16) |
| 2017 | 64.35 (±9.87) | 63.09 (±9.38) | | 66.46 (±10.39) |
| 2018 | 67.67 (±10.37) | 66.52 (±10.37) | | 68.39 (±10.42) |

SD: standard deviation

**Supplementary 5.:** Distribution of patients based on center of origin

| **Center of origin** | **Total (%)** | **SCC**  **(% of total)** | **AC**  **(% of total)** | **P-value** |
| --- | --- | --- | --- | --- |
| Gastroenterology | 373  (100%) | 300 (80.43%) | 73 (19.57%) | <0.001^1^ |
| Surgery | 1141 (100%) | 747 (65.47%) | 394 (34.53%) |  |

SCC: squamous cell cancer, AC: adenocarcinoma; ^1^: Chi-squared test

**Supplementary 6.:** Ratio of males in the two main histological groups

| **Year** | **SCC male (n)** | **SCC male (%)** | **SCC total (n)** | **AC male (n)** | **AC male (%)** | **AC total (n)** |
| --- | --- | --- | --- | --- | --- | --- |
| 1992 | 15 | 83.33% | 18 | 0 |  | 0 |
| 1993 | 20 | 95.24% | 21 | 0 |  | 0 |
| 1994 | 31 | 93.94% | 33 | 4 | 100.00% | 4 |
| 1995 | 16 | 100.00% | 16 | 0 |  | 0 |
| 1996 | 19 | 90.48% | 21 | 1 | 100.00% | 1 |
| 1997 | 32 | 94.12% | 34 | 1 | 100.00% | 1 |
| 1998 | 41 | 83.67% | 49 | 2 | 100.00% | 2 |
| 1999 | 47 | 88.68% | 53 | 5 | 83.33% | 6 |
| 2000 | 25 | 80.65% | 31 | 6 | 85.71% | 7 |
| 2001 | 36 | 83.72% | 43 | 4 | 100.00% | 4 |
| 2002 | 36 | 76.60% | 47 | 12 | 80.00% | 15 |
| 2003 | 36 | 85.71% | 42 | 12 | 100.00% | 12 |
| 2004 | 41 | 91.11% | 45 | 8 | 80.00% | 10 |
| 2005 | 43 | 86.00% | 50 | 14 | 70.00% | 20 |
| 2006 | 40 | 75.47% | 53 | 21 | 91.30% | 23 |
| 2007 | 29 | 82.86% | 35 | 16 | 88.89% | 18 |
| 2008 | 109 | 84.50% | 129 | 36 | 76.60% | 47 |
| 2009 | 92 | 79.31% | 116 | 39 | 78.00% | 50 |
| 2010 | 123 | 83.11% | 148 | 39 | 82.98% | 47 |
| 2011 | 105 | 83.33% | 126 | 47 | 74.60% | 63 |
| 2012 | 102 | 76.12% | 134 | 54 | 84.38% | 64 |
| 2013 | 100 | 79.37% | 126 | 45 | 78.95% | 57 |
| 2014 | 107 | 80.45% | 133 | 51 | 80.95% | 63 |
| 2015 | 110 | 86.61% | 127 | 40 | 85.11% | 47 |
| 2016 | 90 | 78.95% | 114 | 51 | 79.69% | 64 |
| 2017 | 85 | 80.95% | 105 | 49 | 77.78% | 63 |
| 2018 | 20 | 68.97% | 29 | 35 | 76.09% | 46 |
| **Total** | **1550** | **82.53%** | **1878** | **592** | **80.65%** | **734** |

**Supplementary 7.:** Distribution of primary esophageal cancers by year

| **Year** | **SCC**  **(n, %)** | **AC**  **(n, %)** | **Other primary esophageal cancer (n)** | **No data**  **(but primary esophageal cancer, n, %)** | | **Number of cases** | |
| --- | --- | --- | --- | --- | --- | --- | --- |
| 1992 | 18 (100%) | 0 (0%) | 0 (0%) | | 0 (0%) | | 18 |
| 1993 | 21 (91%) | 0 (0%) | 2 (9%) | | 0 (0%) | | 23 |
| 1994 | 33 (89%) | 4 (11%) | 0 (0%) | | 0 (0%) | | 37 |
| 1995 | 16 (100%) | 0 (0%) | 0 (0%) | | 0 (0%) | | 16 |
| 1996 | 21 (95%) | 1 (5%) | 0 (0%) | | 0 (0%) | | 22 |
| 1997 | 34 (97%) | 1 (3%) | 0 (0%) | | 0 (0%) | | 35 |
| 1998 | 49 (96%) | 2 (4%) | 0 (0%) | | 0 (0%) | | 51 |
| 1999 | 53 (90%) | 6 (10%) | 0 (0%) | | 0 (0%) | | 59 |
| 2000 | 32 (67%) | 8 (17%) | 5 (10%) | | 3 (6%) | | 48 |
| 2001 | 43 (81%) | 4 (8%) | 3 (6%) | | 3 (6%) | | 53 |
| 2002 | 49 (72%) | 15 (22%) | 2 (3%) | | 2 (3%) | | 68 |
| 2003 | 43 (74%) | 12 (21%) | 2 (3%) | | 1 (2%) | | 58 |
| 2004 | 47 (80%) | 10 (17%) | 0 (0%) | | 2 (3%) | | 59 |
| 2005 | 50 (63%) | 20 (25%) | 3 (4%) | | 7 (9%) | | 80 |
| 2006 | 53 (61%) | 23(26%) | 1 (1%) | | 10 (11%) | | 87 |
| 2007 | 35 (53%) | 18(27%) | 0 (0%) | | 13 (20%) | | 66 |
| 2008 | 129 (61%) | 48 (23%) | 4 (2%) | | 30 (14%) | | 211 |
| 2009 | 116 (57%) | 50 (25%) | 6 (3%) | | 32 (16%) | | 204 |
| 2010 | 148 (65%) | 47 (21%) | 4 (2%) | | 27 (12%) | | 226 |
| 2011 | 126 (55%) | 63 (28%) | 4 (2%) | | 36 (16%) | | 229 |
| 2012 | 137 (60%) | 64 (28%) | 6 (3%) | | 22 (10%) | | 229 |
| 2013 | 127 (64%) | 57 (29%) | 7 (4%) | | 9 (5%) | | 200 |
| 2014 | 135 (61%) | 64 (29%) | 7 (3%) | | 14 (6%) | | 220 |
| 2015 | 127 (64%) | 47 (24%) | 6 (3%) | | 20 (10%) | | 200 |
| 2016 | 118 (57%) | 64 (31%) | 8 (4%) | | 16 (8 %) | | 206 |
| 2017 | 106 (56%) | 63 (33%) | 9 (5%) | | 11 (6%) | | 189 |
| 2018 | 29 (34%) | 46 (53%) | 0 (0%) | | 11 (13%) | | 86 |
| **Total** | **1895** | **737** | **79 (3%)** | | **269 (9%)** | | **2980** |
